# Unique gene program of rat small resistance mesenteric arteries as revealed by deep RNA sequencing

**DOI:** 10.14814/phy2.12450

**Published:** 2015-07-08

**Authors:** John J Reho, Amol Shetty, Rachael P Dippold, Anup Mahurkar, Steven A Fisher

**Affiliations:** 1 Department of Medicine, Division of Cardiovascular Medicine, University of Maryland-BaltimoreBaltimore, Maryland, 21201; 2 Institute for Genome Sciences, University of Maryland-BaltimoreBaltimore, Maryland, 21201

**Keywords:** Aorta, mesenteric artery, mitochondria, Nkx2-3, vascular smooth muscle

## Abstract

Deep sequencing of RNA samples from rat small mesenteric arteries (MA) and aorta (AO) identified common and unique features of their gene programs. ∼5% of mRNAs were quantitatively differentially expressed in MA versus AO. Unique transcriptional control in MA smooth muscle is suggested by the selective or enriched expression of transcription factors Nkx2-3, HAND2, and Tcf21 (Capsulin). Enrichment in AO of PPAR transcription factors and their target genes of mitochondrial function, lipid metabolism, and oxidative phosphorylation is consistent with slow (oxidative) tonic smooth muscle. In contrast MA was enriched in contractile and calcium channel mRNAs suggestive of components of fast (glycolytic) phasic smooth muscle. Myosin phosphatase regulatory subunit paralogs Mypt1 and p85 were expressed at similar levels, while smooth muscle MLCK was the only such kinase expressed, suggesting functional redundancy of the former but not the latter in accordance with mouse knockout studies. With regard to vaso-regulatory signals, purinergic receptors P2rx1 and P2rx5 were reciprocally expressed in MA versus AO, while the olfactory receptor Olr59 was enriched in MA. Alox15, which generates the EDHF HPETE, was enriched in MA while eNOS was equally expressed, consistent with the greater role of EDHF in the smaller arteries. mRNAs that were not expressed at a level consistent with impugned function include skeletal myogenic factors, IKK2, nonmuscle myosin, and Gnb3. This screening analysis of gene expression in the small mesenteric resistance arteries suggests testable hypotheses regarding unique aspects of small artery function in the regional control of blood flow.

## Introduction

Blood vessels within the vascular system are highly diverse and functionally specialized. Larger elastic arteries are specialized for conduit functions, medium sized arteries for distributive functions, and smaller arteries for providing rapid changes in arterial diameter (resistance) for the acute regulation of blood flow and pressure (Mulvany and Aalkjaer 1990; Chilian 1997; Tanko and Matrougui 2002; Duncker and Bache 2008). Vascular smooth muscle is also phenotypically diverse with contractile properties that parallel those of the blood vessels (reviewed in (Fisher 2010; Reho et al. 2014b)). Smooth muscle of the smaller arteries display phasic (fast) contractile activity, termed vasomotion (Haddock and Hill 2005), and express components of the fast gene program including the fast isoforms of smooth muscle myosin heavy chain (smMHC) (DiSanto et al. 1997) and a unique splice variant of its regulatory enzyme Myosin Phosphatase targeting subunit 1 (Mypt1) (Payne et al. 2004; Zhang and Fisher 2007; Reho et al. 2014a). In contrast large conduit arteries display tonic contractile activity (force maintenance) and express nearly exclusively the slow isoforms of the contractile proteins. Differences between large and small arteries in the expression of ion channels regulating muscle activation have also been reported, as for example the inverse relation between artery size and expression of Kir2.1 (Quayle et al. 1996). Another portion of arterial functional diversity resides within the endothelium (Regan and Aird 2012) and signals that regulate vascular tone. In the larger arteries nitric oxide (NO) generated by NO synthase is the dominant endothelial-derived relaxing factor (EDRF), while in the smaller arteries, endothelial-derived hyperpolarizing factors and their activation of potassium channels play the dominant role (reviewed in (Campbell and Gauthier 2013)).

However, there has been little systematic study of the gene program of the smaller resistance-type arteries, and there remains limited understanding of differences in gene programs that may underlie functional differences in blood vessels throughout the vascular system. This limited understanding in part reflects the difficulty in assaying the gene program in the smaller arteries. Indeed most studies of arterial smooth muscle gene expression and transcriptional control rely on the larger arteries (Owens et al. 2004), and given the differences as described above it is uncertain as to how these findings may apply to smaller arteries. Recent studies have demonstrated the feasibility of determining small muscular artery gene programs using high throughput Deep RNA Sequencing (RNASeq), in which the effect of exercise and obesity on gene expression in rat skeletal muscle small arteries were examined (Jenkins et al. 2014; Padilla et al. 2014). In the current study we took advantage of the rat mesenteric arterial arcade to obtain sufficient sample of small arteries from individual rats to perform a comprehensive and unbiased analysis of gene expression using RNASeq and compared it with that of the thoracic aorta as a reference vessel. RNASeq provides numerous advantages over older methods used for this type of analysis: (1) with moderate intensity sequencing lower abundance mRNAs are captured and nearly all mRNAs within a sample are assayed, (2) data can be reported in absolute terms as number of reads normalized to transcript length facilitating comparisons between gene families and amongst gene family members. Most other methods report relative differences in expression between samples as fold change, and (3) specific family members are identified by their unique sequences, obviating mistakes in determination of expression due to cross-hybridization of probes to related family members. Using this detailed analysis we profiled the gene program of the rat smaller resistance-type mesenteric arteries and contrast it with that of a large conduit artery, the aorta, and discuss how the gene program may underlie the unique functional properties of the smaller arteries.

## Methods

### Blood vessels

Adult male Sprague–Dawley rats (∼250 g; 12 weeks old) were obtained from Harlan Laboratories (Indianapolis, IN) and handled in accordance with IACUC guidelines at the University of Maryland School of Medicine. Rats were euthanized by CO_2_ inhalation followed by cervical dislocation. Mesenteric arteries and thoracic aortas were quickly dissected and preserved in RNA Later (Qiagen, Valencia, CA). In more detail, from one male rat the thoracic aorta (Sample AO1) was removed followed by the removal of the small intestines and each submerged into RNA Later. The mesenteric arterial arcade, excluding the superior mesenteric artery, was then dissected, cleaned of fat, and submerged in RNA Later and stored at 4°C prior to processing (Sample MA1). The thoracic aorta was similarly processed from a second male rat (Sample AO2) and the mesenteric arterial arcade from a third male rat (Sample MA2). Tissues were homogenized using disposable plastic pestles (Kimble Chase) and total RNA was column purified (RNEasy Mini Kit) according to the manufacturer’s instruction (Qiagen) and eluted in 30 *μ*L volume. RNA yields were as follows: Sample AO1: 4.4 *μ*g; Sample AO2: 2.1 *μ*g; Sample MA1: 1.4 *μ*g; Sample MA2: 2.2 *μ*g. RNA quality was examined and A260/280 ratios were above 2.0 for all samples.

### Processing and sequencing of RNA

RNA sequencing was performed at the Genome Resource Center at the Institute for Genome Sciences at the University of Maryland Baltimore. mRNA-Seq libraries were generated from the four samples using the TruSeq RNA Sample Prep Kit (Illumina, San Diego, CA) as per the manufacturer’s protocol. The four libraries were multiplexed on a single lane. The libraries were sequenced on the Illumina Hiseq 2000 sequencing instrument using a 101 bp paired-end protocol to generate an average of 94.9 million reads per library. The raw data from the sequencer used the Illumina CASAVA pipeline which consists of image analysis, base-calling and de-multiplexing to generate the sequencing reads in FastQ format files. FastQC and in-house pipelines were utilized to assess sequence quality and determine if additional trimming was necessary.

The 101 bp sequence reads were aligned to the R. *norvegicus* (Rnor5.72) reference genomic sequence (Ensembl) using the TopHat read alignment tool (Trapnell et al. 2009, 2012). TopHat aligns RNA-Seq reads to mammalian-sized genomes using the ultra-high-throughput short read aligner Bowtie (Langmead et al. 2009), and then analyzes the mapping results to identify splice junctions between exons. The output from TopHat is obtained as BAM format describing individual read alignments within the reference genome and the splicing information of each read. In the alignment phase, we allowed up to two mismatches per 30 bp segment and discarded reads that aligned to more than 2 locations. The alignment files obtained from the TopHat alignment tool were analyzed to generate the alignment statistics for each sample including total number of reads, number of mapped reads and percent of reads mapped to the reference genome. Read counts for each annotated gene were computed and normalized by mRNA length and the library size to generate counts per kilobase per million reads (kpm) for each gene. These normalized values were used to compute the correlation between replicate samples and for principal component analysis of all samples.

### Statistical analysis of RNASeq data

The read counts for each gene were calculated using HTSeq (Anders et al. 2015) and then provided as input for the DESeq (Anders and Huber 2010), an R statistical package. Within DESeq, the raw read counts for each gene were used to remove genes with no or low expression (i.e., <10 reads across all samples). The raw read count for the remaining genes were utilized to estimate the size factors for each sample in order to normalize the counts from different samples that may have been sequenced at different depths rendering them comparable. As a simple Poisson distribution (i.e., mean = variance = *λ*) does not account for the overdispersion observed within RNA-Seq read counts due to biological variation, the read count data were modeled using the negative binomial (NB) distribution which involves separate parameters for the mean and variance for each gene within each condition. The normalized read counts were further utilized to fit the NB model and compute the mean and variance of the expression for each gene for the AO and MA conditions. The mean and variance estimates for each gene were further used to test for the null hypothesis, that is, the mean expression of the gene is the same in both conditions, using a negative binomial test analogous to the Fisher’s exact test. Next, we contrasted the two AO samples against the two MA samples and computed a fold-change difference between the two conditions as well as a *P*-value associated with each of the expressed genes using the NB test. In order to adjust for multiple testing and reduce the type I error, that is false positive detection rate, the *P*-value for each gene was adjusted using the Benjamini–Hochberg method and their respective False Discovery Rates (FDR) were computed. The FDR values estimate the probability of the differential expression of the gene being detected by chance.

The list of differentially expressed genes was filtered on several criteria. First, genes with <10 normalized read counts in each replicate sample were filtered out. Additionally, only genes showing lower estimates of false discovery, that is FDR ≤0.05, and greater than twofold difference in expression values between the AO and MA were retained. In order to account for the higher variance in the MA replicates as observed from the principal component analysis, we further filtered the list of significantly differentially expressed genes to ensure that the direction of fold change was consistent for both the AO samples against the individual MA1 and MA2 samples. Additionally, the ratio of the normalized expression between the MA1 and MA2 samples were computed and only differentially expressed genes with values within 2 standard deviations of the mean (*μ *= 1) were retained in the final list of significantly differentially expressed genes. The final list of differentially expressed genes was used to compute the enrichment of biological pathways separately for the upregulated and downregulated genes using tools such as DAVID (da Huang et al. 2009) and Ingenuity Pathway Analysis (IPA). The full data set is available at: http://www.ncbi.nlm.nih.gov/geo/query/acc.cgi?token=wpevuemixtuvxmr&acc=GSE64450

### Validation of RNASeq

Differential expression of a select subset of genes of interest was validated by real-time PCR (StepOnePlus, Applied Biosystems, Foster City, CA) of mRNAs from MA and AO samples obtained from three additional adult male Sprague–Dawley rats. qPCR was performed with predesigned Taqman probes and a Fast Advanced Taqman Master Mix (Applied Biosystems) or by Fast SYBR Green PCR Master Mix (Applied Biosystems) and primers designed using Primer3plus software. Data were analyzed using the comparative Ct method and expressed as fold change. To determine the relative expression of the mRNA in the different cell types within the blood vessel, qPCR was performed on RNAs purified from endothelial versus mural fractions of rat small mesenteric arteries (generously provided by Dr. An Huang (Sun et al. 2011)).

## Results

### RNA sequencing

**Figure 1:**
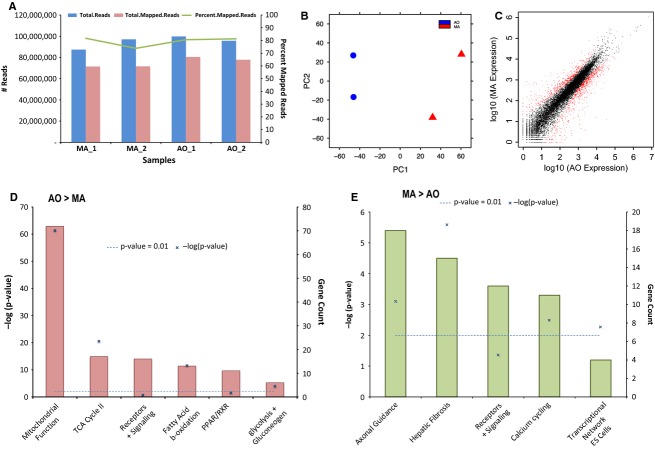
Differential gene expression in conduit (AO) versus resistance arteries (MA). (A) RNA-Seq read depth and mapped reads for aorta and mesenteric artery samples (*n* = 2). (B) Principal component analysis (PCA) demonstrates separation of groups on principal component 1. (C) Group average normalized read counts (log2) for all genes mapped above the background cutoff (19,974 genes with a cutoff of 10 read counts) are plotted with differentially expressed genes highlighted in red. Differentially expressed genes (DEGs) were determined by DESeq with FDR <0.05 and fold change >2 and a within group criteria of 1.25 normalized expression ratio. (D–E) Processes with an enrichment of DEGs were determined for each group (AO > MA and MA > AO, respectively) using gene ontology enrichment analysis (DAVID; david.abcc.ncifcrf.gov) against the background of all genes mapped in the study.

**Figure 2:**
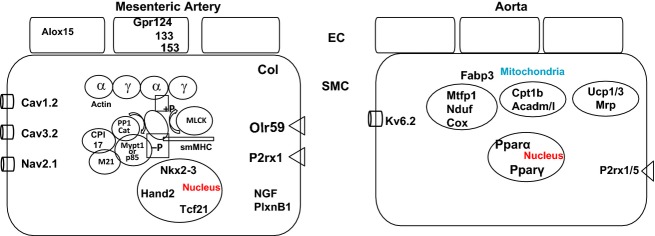
Schematic overview of differentially expressed genes in rat mesenteric arteries versus aorta as determined by RNASeq. Some of the mRNAs that are enriched in each tissue are shown. The assignment of cell type is based on qPCR validation or prior studies and is not definitive. The mesenteric artery is enriched in transcription factors (Nkx2-3, Hand2, Tcf21), contractile proteins and specifically *γ*-actin, calcium channels (Cav1.2, 3.2) and the sodium channel (Nav2.1), the purinergic (P2rx1) and the olfactory receptor (Olr59). Alox15 and the orphan G protein-coupled receptors (Gpr124, 133, 153). The aorta is enriched transcription factors (Ppar-*α*/*γ*) and mRNAs ecndoing proteins of mitochondria and fatty acid metabolism as well as a potassium channel (Kv6.2).

Eighty to one hundred million reads were generated from each MA and AO sample with 70–80% mapped to the genome (Fig.1A). The MA samples were significantly different from the AO samples (Fig.1B) while the variability between MA samples from different rats was greater than that of the AO samples, likely reflecting variability introduced by the tissue dissections and small size of the samples. 19,974 genes were expressed above the cutoff for background of 10 read counts. Of these 942 genes’ mRNAs (∼5%) were ≥2-fold different between MA and AO with 305 enriched in MA and 637 enriched in AO (Fig.1C). According to DAVID and IPA pathway analyses functional groups of genes especially enriched in AO included those coding for mitochondrial proteins, oxidative phosphorylation, fatty acid metabolism, and glycolysis and gluconeogenesis (Fig.1D). The most highly represented gene families in the grouping of mitochondrial proteins included NADH dehydrogenases (Nduf, *n* = 32), mitochondrial ribosomal proteins (Mrp, *n* = 18) and Cytochrome C Oxidases (Cox, *n* = 10). These mRNAs were 3–8 fold enriched in AO. There were fewer genes clustered in MA, which included those for axonal guidance, hepatic fibrosis, calcium cycling, and transcriptional networks (ES cells) (Figs.1E, 2). The most highly represented gene families in the grouping of hepatic fibrosis were the collagens (Col, *n* = 10) while in the category of axonal guidance were members of different gene families including NGF, PlexinB1, EphrinB3, Semaphorin6C, and L1CAM. A substantial number of mRNAs in the categories of receptors and signaling were also differentially expressed in MA and AO, but when grouped together these fell below the cut-off for statistical significance.

### Transcription factors

Several transcription factors mRNAs were (1) enriched in MA, and (2) relatively abundant in MA, using Myocardin (Myocd), a member of the MRTF family and critical regulator of smooth muscle differentiation (Pipes et al. 2006; Parmacek 2007) as a reference (Table1). Nkx2-3, a member of the NK class of homeodomain containing transcription factors (Akazawa and Komuro 2005; Bartlett et al. 2010; Reamon-Buettner and Borlak 2010) was highly enriched in MA, with expression levels higher than Myocd, while in AO this ratio was reversed. Myoc was also enriched in MA versus AO but to a much lesser degree. None of the other 14 Nkx family members were significantly expressed in either tissue (data not shown). The basic helix-loop-helix (bHLH) transcription factors, Tcf21 (transcription factor 21, also known as epicardin or Pod1) and HAND2, were also highly enriched in MA versus AO but to a lesser magnitude than Nkx2-3, while HAND1 was not expressed in either tissue (data not shown). Of note Nkx2-3, Tcf21, and HAND2 were amongst the 10 most highly enriched mRNAs in MA versus AO. The Islet1 (Isl1) homeobox mRNA was the transcription factor most highly enriched in MA versus AO resulting from low expression in MA versus nearly undetectable levels in AO. The classic homeobox genes (Hoxa-d) involved in segmentation and patterning of tissues were present in AO and MA but were not differentially expressed (data not shown). Notable amongst transcription factors enriched in AO were the peroxisome proliferative activated receptors PPAR*α* and *γ*, critical regulators of transcription of genes involved in fatty acid metabolism (Madrazo and Kelly 2008).

**Table 1:** Transcription factor mRNAs in MA and AO in Rpkm, reads per kilobase of transcript per million mapped reads.

Gene	Common Name	MA		AO		MA avg	AO avg	MA/AO	FDR
		1	2	1	2				
Nkx2-3	NK2 homeobox 3	52	54	0.7	0.5	53	0.6	89	2.45e-24
Myocd	Myocardin	6.7	27	1.7	4.1	16	2.9	5.8	0.0007
Isl1	ISL LIM homeobox 1	4.1	9.4	0	0.1	6.8	0.05	135	2.75e-7
Sox10	SRY-box 10	6.7	11	2.3	1.4	9.0	1.9	4.7	0.03
Tbx18	T-box 18	0.8	0.9	1.6	3.0	0.85	2.3	0.37	0.009
Ppar*α*	Peroxisome proliferator-activated receptor alpha	2.3	2.2	14	15	2.25	15	0.15	1.24e-9
Ppar*γ*	Peroxisome proliferator-activated receptor gamma	45	19	89	60	32	74	0.43	0.0005
Tcf21	Transcript factor 21	31	20	1.1	0.9	25	1.0	25	8.80e-10
Trerf1	Transcriptional-regulating factor 1	5.8	4.9	0.9	1.2	5.4	1.1	4.9	0.04
Hand2	Heart and neural crest derivates expressed 2	33	98	1.2	4.2	65	2.7	24	3.96e-8

FDR, false discovery rate.

### Contractile Regulatory enzymes: Phosphatases and kinases

Myosin light chain kinase (MLCK) and phosphatase (MP) enzymes are primary mediators of smooth muscle contraction and relaxation, respectively (reviewed in (Fisher 2010; Ito et al. 2004)). Myosin light chain kinase (Mylk) and MP regulatory subunit (Mypt1 =  PPP1R12a) were 5–7-fold enriched in MA (Table2), consistent with prior studies (Gong et al. 1992; Payne et al. 2006; Gao et al. 2013) though the difference in MLCK did not reach statistical significance. PPP1R12c (p85), the third and less studied member of the MP regulatory subunit family, was expressed at modestly higher levels than Mypt1 in AO and MA, with a similar enrichment in MA that did not reach statistical significance. The second Mypt family member, PPP1R12b (Mypt2), generated a short mRNA from an internal transcription start site, as expected (Chen et al. 1994; Dippold and Fisher 2014a), coding for the small (M21) subunit of MP. Like the other MP subunits it tended to be more highly expressed in MA versus AO. Full length Mypt2 mRNA was not detected consistent with its selective transcription in striated muscle (Fujioka et al. 1998). All three members of the Type1 serine-threonine phosphatase catalytic subunit family (PPP1c a-c) were abundant in MA and AO with PPP1c-b, the catalytic subunit of MP, modestly more highly expressed and tending to be more abundant in MA. Amongst the four family members of the inhibitory subunit of MP (PPP1R14a-d), only CPI-17 (PPP1R14a) and PHI-1 (PPP1R14b) were significantly expressed in MA and AO. CPI-17 was much more abundant than PHI-1 in MA while this difference was narrowed in AO, and like other MP subunits, CPI-17 tended to be more abundant in MA versus AO. In contrast to MP subunits, Mylk was the only abundant MLCK mRNA in smooth muscle as Mylk2 (skeletal/cardiac) and Mylk3 were minimally detected (data not shown). IKK2 (IKBKB) has been suggested to function as a smooth muscle myosin light chain kinase (Ying et al. 2013), but it was ∼10 and ∼30 fold less abundant than Mylk in AO and MA, respectively.

**Table 2:** Phosphatase and kinase mRNAs in MA and AO in Rpkm, reads per kilobase of transcript per million mapped reads.

Gene	Common Name	MA		AO		MA avg	AO avg	MA/AO	FDR
		1	2	1	2				
Ppp1r12a	Mypt1	69	180	19	26	125	22	5.6	0.006
Ppp1r12b	Mypt2(M21)	143	214	21	45	179	33	5.4	ND
Ppp1r12c	P85	224	315	49	69	270	58	4.6	ND
Ppp1c-a	PP1alpha	134	131	101	94	132	98	1.4	ND
Ppp1c-b	PP1beta	160	194	52	67	177	60	3.0	ND
Ppp1c-c	PP1 gamma	64	81	44	42	73	43	1.7	ND
Ppp1r14a	CPI-17	307	472	66	180	389	123	3.2	ND
Ppp1r14b	PHI-1	30	43	44	37	37	40	0.9	ND
Mylk	MLCK	245	1016	68	106	631	87	7.3	ND
Ikbkb	IKK2	22	22	8.6	10	22	9.4	2.3	ND
Ppp1r3b	Glycogen-targeting subunit B	3.6	9.6	37	24	6.6	30	0.2	6.33e-8
Ppp1r3d	Glycogen-targeting subunit D	16	24	5.7	4.1	20	4.9	4.1	0.03

FDR, false discovery rate; ND, Not differentially expressed.

Diversity in the serine-threonine phosphatases is generated by a variety of regulatory (targeting) and inhibitory subunits partnering with the three catalytic subunits (Ceulemans and Bollen 2004). PPP1R3, the glycogen-targeting subunit family (GlPP1G), provides an interesting example of complementary and tissue-specific expression. PPP1R3b was ∼5-fold enriched in AO while PPP1R3d was ∼4-fold enriched in MA. PPP1R3c was equally expressed in MA and AO at levels similar to the other subunits (data not shown). Other PPP1-6 family subunit mRNAs were present in AO and MA without differential expression (data not shown).

### Contractile machinery

Smooth muscle myosin heavy chain (Myh11) is a highly specific marker of smooth muscle. It was one of the most abundant mRNAs in MA and ∼90-fold more abundant than nonmuscle myosin heavy chain-B (Myh10) (Table3). Myh11 and smooth muscle *α*-actin (Acta2) tended to be enriched in MA though neither reached statistical significance. In contrast, smooth muscle *γ*-actin (Actg2) was highly enriched in MA, where it was expressed at a level modestly less than Acta2, while in AO it was expressed at low levels compared to Acta2. Most of the other contractile mRNAs followed a general trend of being twofold or less enriched in MA, including calponin, caldesmon, actinin, and tropomyosin (data not shown).

**Table 3:** Contractile mRNAs in MA and AO in Rpkm, reads per kilobase of transcript per million mapped reads.

Gene	Common Name	MA		AO		MA avg	AO avg	MA/AO	FDR
		1	2	1	2				
Myh11	smMHC	858	2087	121	239	1472	180	8.2	ND
Myh9	NM MHC-A	0.3	0.005	0.2	0.2	0.2	0.2	1.0	ND
Myh10	NM MHC-B	12	21	4.4	25	17	15	1.1	ND
Acta2	SM alpha actin	3434	6815	775	2380	5124	1578	3.2	ND
Actg2	SM gamma actin	883	3940	77	64	2411	70	34	2.0e-5
Actb	Beta-actin	1374	2644	769	888	2009	829	2.4	ND

FDR, false discovery rate; ND, Not differentially expressed.

### Ion channels and transporters

Several of the subunits of the L- (Cacna1c, b2, b3) and T- (Cacna1g, h) type calcium channels were enriched in MA, while none were enriched in AO (Table4). Amongst the potassium channels, the voltage-gated potassium channel beta-1 subunit (Kcnab1) mRNA was enriched in MA while KCNg2 and KCNk3 were enriched in AO. KCNm-a1 and -b1 subunits of BKCa channel tended to be enriched in MA but did not reach statistical significance, while b2-4 subunit mRNAs were not expressed to any appreciable level (data not shown). The inward rectifying potassium channel encoded by Kir2.1 (Kcnj2) was expressed at low and similar levels in MA and AO (data not shown). Kcnj5 (G protein activated) and Kcnj8 (ATP sensitive) were the most abundant members of the Kcnj (Kir) family with similar levels of expression in MA and AO (data not shown). Hyperpolarization and cyclic-nucleotide-gated (HCN1-4) family of potassium channels were detected at low levels in MA and AO (data not shown). Amongst the sodium channel subunits SCN1b (data not shown) and SCN 7a were by far the family members most highly expressed with the latter enriched in MA.

**Table 4:** Ion Channels and Transporters mRNAs in MA and AO in Rpkm, reads per kilobase of transcript per million mapped reads.

Gene	Common Name	MA		AO		MA avg	AO avg	MA/AO	FDR
		1	2	1	2				
Cacna1c	LTCC, a1c subunit	4.5	16	1.6	2.0	10	1.8	5.6	0.0008
Cacnb2	LTCC, b2 subunit	4.4	15	1.7	2.2	9.7	2.0	4.8	0.007
Cacnb3	LTCC, b3 subunit	7.2	22	1.7	3.1	15	2.4	6.3	0.001
Cacna1g	Cav3.1 subunit	3.7	8.4	1.7	1.2	6.1	1.5	4.1	0.04
Cacna1h	Cav3.2 subunit	6.9	8.2	1.5	0.9	7.6	1.2	6.3	0.0005
Kcnab1	Kvb1.3	6.6	6.2	1.5	1.0	6.4	1.3	4.9	0.03
Kcng2	Kv6.2	1.2	1.5	5.4	11	1.4	8.2	0.2	1.03e-6
Kcnk3	K2p3.1	19	23	61	46	21	54	0.4	0.0002
Kcnma1	BKCa, a subunit	9.7	14	1.3	6.5	12	3.9	3.1	ND
Kcnmb1	BKCa, b1subunit	83	160	13	58	122	36	3.4	ND
Scn7a	Nav2.1	25	36	5.5	4.6	31	5.1	6.1	0.0006

FDR, false discovery rate; ND, Not differentially expressed; LTCC, L-type Calcium Channel; BKCa, large conductance calcium activated potassium channel.

### Metabolism

Many of the mRNAs encoding for proteins of mitochondrial function, oxidative phosphorylation and fatty acid metabolism were amongst the 50 most highly enriched mRNAs in AO (Table5). These include Cpt1b, the muscle isoform of Carnitine O-palmitoyltransferase1, FABP3, fatty acid-binding protein heart, and Acadm and AcadL, medium and long chain AcyCoA dehydrogenase. Of all of the mRNAs with at least moderate expression, the mitochondrial uncoupling protein UCP1 was most highly enriched in AO as it was just slightly above background in MA and highly expressed in AO. The related family member UCP3 had lower expression but with a similar pattern, while the third family member UCP2 was also expressed at lower levels and more modestly enriched in MA. Other metabolic mitochondrial and metabolic genes that were greater than 20-fold enriched in AO included Gpld1, Akr1cl, Gk, Ccrn4l, Gpd2, Acacb, and Mtfp1 (data not shown).

**Table 5:** Metabolic and mitochondrial mRNAs in MA and AO in Rpkm, reads per kilobase of transcript per million mapped reads.

Gene	Common Name	MA		AO		MA avg	AO avg	MA/AO	FDR
		1	2	1	2				
Cpt1b	Carnitine palmitoyltransferase 1B	4.6	6.2	153	160	5.4	157	0.03	2.77e-17
Cs	Citrate synthase	121	138	278	340	130	309	0.4	0.01
Cyp2e1	Cytochrome P450 2E	1.1	0.2	41	24	0.7	33	0.02	ND
Cyp4b1	Cytochrome P450 4B	7.6	3.9	20	15	5.8	18	0.3	1.27e-5
Cyp4f18	Cytochrome P450 F18	4.7	8.2	0.7	2.5	6.5	1.6	4.1	ND
Fabp3	Fatty acid-binding protein 3	11	17	167	406	14	287	0.05	9.01e-20
Acadm	Acyl-CoA dehydrogenase, Medium	64	60	509	611	62	560	0.11	3.22e-6
AcadL	Acyl-CoA dehydrogenase, Long	96	91	629	844	94	737	0.13	1.14e-5
UCP1	Uncoupling protein 1	0.4	0.3	708	649	0.35	679	5.2e-4	1.45e-34
UCP2	Uncoupling protein 2	105	81	30	23	93	27	3.4	ND
UCP3	Uncoupling protein 3	2.0	3.1	27	28	2.6	28	0.09	1.99e-15

FDR, false discovery rate; ND, Not differentially expressed.

One concern was that high enrichment of mitochondrial and metabolic mRNAs in AO could represent differential contamination of the samples by fat (adipocytes). This does not appear to be the case as the adipocyte marker Resistin was only ∼1.5-fold and FABP4, fatty acid-binding protein 4, adipocyte isoform, ∼3-fold more abundant in AO versus MA (data not shown). In contrast, as noted above FABP3, the muscle isoform, was 20-fold more abundant in AO versus MA, and other mitochondrial and metabolic mRNAs were 30–200-fold enriched in AO versus MA.

### Vaso-active signaling

Force is activated by sympathetic signaling through purinergic, *α*-adrenergic and Neuropeptide Y (NPY) receptors expressed in arterial smooth muscle (Wier et al. 2009). Amongst the purinergic receptors mediating vasoconstriction (P2rx), P2rx1 was by far the most abundant and enriched in MA, while P2rx5 was expressed at much lower levels and reciprocally enriched in AO resulting from its low expression in MA (Table6). P2rx1 and P2rx5 were expressed at identical levels in AO. P2ry1 and P2ry2, the endothelial purinergic receptors, were present at similar levels in MA and AO, while other members of the P2r family were present at lower levels (P2rx4) or not detectable (data not shown). Npy1R was the only member of this family expressed in these arteries and was enriched in MA. Amongst the *α*-adrenergic receptors Adra1a+ d were most abundant with no significant difference between or amongst groups and lower expression of Adra2x family members. Amongst *β*-receptors, mediators of vasorelaxation, *β*1, 2 were of low abundance (data not shown) while *β*3-adrenergic was more abundant and modestly enriched in AO.

**Table 6:** Signaling and receptor mRNAs in MA and AO in Rpkm, reads per kilobase of transcript per million mapped reads.

Gene	Common Name	MA		AO		MA avg	AO avg	MA/AO	FDR
Npy1r	Neuropeptide Y1 receptor	22	27	7.1	2.1	25	4.6	5.4	0.003
P2rx1	Purinergic X1 receptor	198	259	15	31	229	23	9.9	2.94e-5
P2rx5	Purinergic X5 receptor	5.5	4.5	20	24	5	22	0.2	4.48e-8
Adra1a	*α*1a-adrenergic receptor	4.8	15	4.6	4.7	9.9	4.7	2.1	ND
Adra1d	*α*1d-adrenergic receptor	8.6	10	6.8	16	9.3	11.4	0.8	ND
Adrb3	*β*3-adrenergic receptor	34	13	49	36	24	43	0.6	0.006
Alox15	Arachidonate 15-lipoxygenase	36	17	1.1	0.8	27	1.0	27	2.66e-13
Nos1	Neuronal NOS	1.5	0.1	0.2	0.2	0.8	0.2	4	ND
Nos2	Inducible NOS	0.01	0.02	0.2	0.06	0.02	0.13	0.2	ND
Nos3	Endothelial NOS	48	18	24	12	33	18	1.8	ND
Gpr124	G protein-coupled receptor A2	29	67	7.5	9.7	48	8.6	5.6	0.001
Gpr133	G protein-coupled receptor D1	7.0	14	1.6	2.4	11	2.0	5.5	0.01
Gpr153	G protein-coupled receptor 153	11	16	2.0	3.6	14	2.8	5.0	0.01
Olr59	Olfactory receptor 59	5.8	12	1.0	0.5	8.9	0.8	11	1.83e-5

FDR, false discovery rate; ND, Not differentially expressed.

Amongst signals mediating vasodilation mRNAs for endothelial nitric oxide synthase (NOS3) were equally abundant in MA and AO, while the neuronal isoform (NOS1) was less abundant and also not differentially expressed. In contrast Alox15 (Arachidonate 12-lipoxygenase), which converts arachidonic acid to HEETAs and THETAs, endothelial-derived hyperpolarizing factors (Campbell and Gauthier 2013) was minimally expressed in AO and expressed at a level similar to eNOS in MA for almost 30-fold enrichment in MA. Six other members of the Alox family were present at low or undetectable levels in both arteries (data not shown). Members of the Cytochrome P450 family (Cyp) generate the second major class of EDHFs by oxidation of arachidonic acid to EETs (reviewed in (Pfister et al. 2010)). The Cyps are a large family involved in many mono-oxidation reactions, for example, detoxification of drugs. Many Cyps were expressed in MA and AO and a few were also differentially expressed (data not shown).

### Orphan and miscellaneous other receptors

Deep sequencing of the RNA pool could identify receptors not classically associated with vascular function that could represent novel drug targets. Consistent with a recent study in mice (Pluznick et al. 2013), the rat olfactory receptor Olr59 was expressed at moderate levels in MA and ∼10-fold enriched in MA (Table6). The olfactory receptors (Olfr, Olf) comprise over 1000 genes. Olr63 was the only other such receptor expressed at a level similar to that of Olr59 but without differential expression (data not shown). A number of orphan G Protein-coupled receptors (GPRs) were expressed in MA and AO with GPRs 124,133, and 153 most highly enriched in MA (∼5-fold). Other GPRs were highly expressed but at equivalent levels, for example GPR116 and GPR107 (data not shown).

### qPCR Validation

We used qPCR to validate the differential expression of a select subset of mRNAs of particular interest in this study using an independent set of samples (*n* = 3) (Tables7 and 8). In general there was good agreement between the fold change between MA and AO determined by RNASeq and qPCR. The fold change determined by qPCR in some instances was greater than that determined by RNASeq, perhaps reflecting the logarithmic amplification of signal with each cycle. To determine the cell type in the blood vessel wall from which the mRNAs are expressed, we performed qPCR on cDNAs derived from endothelial versus mural fractions as described in Methods. The enrichment of eNOS in the endothelial cell fraction and *α*SMA in the mural fraction confirmed the validity of these fractions (Table8) consistent with our prior study (Zheng et al. 2015). The transcription factor Nkx2-3 was similarly expressed in endothelial and smooth muscle cells. MP subunits Mypt1 and CPI-17 were enriched in the mural fraction. The olfactory receptor Olr59 was highly enriched in the mural fraction.

**Table 7:** qPCR validation of differential expression of select mRNAs in MA versus AO shown as average fold difference (*n* = 3) versus data from RNASeq.

Common Name	RNASeq MA/Ao	qPCR MA/AO
GAPDH	0.6	0.7
Nkx2-3	89	197
Mypt1	5.6	9.6
Ppp1cb	3.0	1.6
PHI-1	0.9	1.2
MLCK	7.3	4.3
smMHC	8.2	5.7
Olr59	11	29

**Table 8:** qPCR assay of select mRNAs in mural vascular smooth muscle (VSM) versus endothelial cell (EC) fractions of mesenteric arteries reported as fold difference.

Common name	qPCR VSM/EC
Nkx2-3	1.6
Olr59	17261
Mypt1	24
CPI-17	3.7
eNOS	0.2
*α*-actin	14

## Discussion

As gene expression is a prerequisite for gene function, this screening study of mesenteric arterial gene expression provides a resource for including or excluding genes in the generation of hypotheses regarding the control of mesenteric resistance artery function. This full data set has been deposited in a publically available web site and should be of substantial utility to vascular biologists, not only in generating hypotheses regarding genes that are expressed, but also excluding a role for genes that are expressed at low levels or not at all. We next consider the functional implications of the gene program of the mesenteric arteries as identified by RNASeq. mRNA levels must be confirmed at the level of protein expression and activity. The correlation between mRNA and protein expression and activity is not exact though the nature of this relationship is still debated (Vogel and Marcotte 2012; Wilhelm et al. 2014; Jovanovic et al. 2015) and has not been determined in a comprehensive fashion in blood vessels.

### MA transcriptional programming

Given the importance of resistance-type arterial smooth muscle in the regulation of blood flow and pressure it is surprising how little is known about its transcriptional control (see for example (Gaengel et al. 2009; Sequeira-Lopez et al. 2015)). Nkx2-3, HAND2 and Tcf21 were ∼25–100-fold enriched in MA, and expressed at similar or greater levels as compared to the ∼5-fold enrichment of the well-characterized muscle differentiation SRF co-factor Myocardin (Pipes et al. 2006; Parmacek 2007). Given the roles of the closely related Nkx paralogue Nkx2-5 and HAND bHLH proteins in cardiac muscle differentiation (Bartlett et al. 2010; Reamon-Buettner and Borlak 2010), it is reasonable to hypothesize that these factors direct the unique gene program of the MA smooth muscle. Nkx2-3 is a member of the Nk (N,K- names of discoverers) class of homeodomain containing transcription factors (reviewed in (Akazawa and Komuro 2005)). Nkx2-3 amongst the 14 Nkx family members has been the subject of limited investigation. Prior studies of Nkx2-3 showed its expression in mesodermal derivatives in the gut (Pabst et al. 1997). Germline knock out of Nkx2-3 causes gut, splenic, vascular, and lymphocytic defects (Pabst et al. 1999; Kellermayer et al. 2011) for which specific mechanisms remain to be determined. The HAND genes (1 + 2) were originally defined by their roles in the development of the myocardium and were subsequently shown to direct gene programs in a variety of cell types (reviewed in (Vincentz et al. 2011)). HAND2 (dHAND) is expressed in vascular mesenchyme and endothelium at the early stages of development; germline inactivation caused early embryonic lethality (E9.5) (Yamagishi et al. 2000). Tcf21 was originally cloned as Capsulin denoting its expression in epicardial progenitors and mesenchyme of visceral organs (Lu et al. 1998). It was also named Pod1 and epicardin and has been proposed to play a positive or negative role in the differentiation of vascular smooth muscle and kidney podocytes (Funato et al. 2003; Plotkin and Mudunuri 2008; Braitsch et al. 2012; Harel et al. 2012; Moncaut et al. 2012). The hypothesis that these transcription factors uniquely program the resistance artery smooth muscle will best be tested using cell-type-specific conditional knockout approaches.

In contrast the PPAR transcription factors (*α*,*γ*), members of the nuclear hormone receptor family with fatty acids as ligands, were enriched in AO. PPARs are critical transcriptional activators of genes required for fatty acid metabolism (Madrazo and Kelly 2008). The enrichment of PPAR in AO correlates with the increased expression of genes of fatty acid metabolism (discussed below). This supports the paradigm of AO as an oxidative (slow) and MA as a glycolytic (fast or mixed) type smooth muscle analogous to oxidative versus glycolytic metabolism in slow (red) versus fast (white) skeletal muscle (Madrazo and Kelly 2008). There has been some study of PPAR(g)s in smooth muscle in relation to vascular contractile function (Carrillo-Sepulveda et al. 2014) but there remains limited understanding of its functional role in conduit versus resistance artery smooth muscle.

This RNASeq data provide an interesting parallel with studies in the simpler and genetically more tractable worm *C. elegans*, which has sixteen nonstriated egg-laying muscles consisting of four types with varying structure and function. An RNAi screen (Hale et al. 2014) identified signaling pathways and transcription factors playing a role in the diversification of these muscles including orthologs of Nkx, Meis, MyoD (bHLH), Tbx1, Six2, and C2H2 zinc finger family, as well as the Notch signaling pathway. Many of the orthologues were identified in the current study as differentially expressed between AO and MA (Table1). Testing the role of these factors in nonstriated muscle diversification may be more efficacious in simpler and genetically more tractable organisms such as the worm or fly. In the mammalian vascular system, the study of unique arterial transcriptional control mechanisms requires identifying gene targets that are uniquely expressed or highly enriched. One such target in MA is smooth muscle-specific *γ*-actin, unique amongst the actin family in its ∼30-fold enrichment in MA. Interestingly a prior study identified a 36 bp enhancer in *γ*-actin that bound Nk-2, SRF and MEF-2 during intestinal smooth muscle differentiation (Phiel et al. 2001). Other genes that are significantly enriched in MA and could be tested as targets of these unique transcriptional control mechanisms include P2rx1 and Olr59. In AO the genes of fatty acid metabolism targeted by PPAR could serve this purpose.

### Contractile properties and metabolism

Smooth muscle myosin and its regulators MLCK and MLCP (subunits) were 3–8 fold more abundant in MA than AO, consistent with prior studies showing several-fold greater expression and activity in phasic versus tonic smooth muscles including vascular tissues (Gong et al. 1992; Payne et al. 2006; Gao et al. 2013) (reviewed in (Fisher 2010)). Both MP regulatory subunit family members Mypt1 (PPP1R12a) and p85 (PPP1R12c) were highly expressed in MA with the latter ∼2-fold more abundant. Mypt1 is classically associated with MP activity in smooth muscle (Alessi et al. 1992) (reviewed in (Hartshorne et al. 2004)), while p85 has been little studied (reviewed in (Dippold and Fisher 2014a)). Functional redundancy between these paralogs could explain the relatively minor effect of smooth muscle-specific knock out of Mypt1 on smooth muscle function (He et al. 2013). The mRNA for the small subunit of MP (M21) originates from an internal transcriptional start site within PPP1R12b (Chen et al. 1994; Dippold and Fisher 2014a) and was expressed at a level similar to the regulatory PPP1r12a+c subunits suggesting that it may function as a third subunit for holoenzymes containing each of the regulatory subunits. In contrast to the MP only a single gene, Mylk1, generated the MLCK mRNA in AO and MA, with no significant expression of MYLK2 or 3. This is consistent with near-complete suppression of arterial smooth muscle force generation by smooth muscle-specific knock out of MLCK (Gao et al. 2013).

As a group, mitochondrial, oxidative, and fatty acid metabolic mRNAs were the most highly differentially expressed with as much as 20–100 fold enrichment in AO. The enrichment of muscle isoforms of Cpt1b, FABP and MCAD, LCAD in AO versus MA is consistent with AO functioning as a slow muscle type with greater reliance on energy from fatty acid metabolism for sustained (tonic) contractions (see (Lynch and Paul 1985; Paul 1989)). The difference is even more striking when considered in relation to the expression of ATP-utilizing enzymes such as myosin and its regulatory kinase, which were 6–8 fold higher in MA. According to our and other’s (Park et al. 2014) review of the literature, there has been very limited study of mitochondrial metabolism in the function of the small resistance-type arteries. Interestingly in the recent study, mitochondrial respiration of human small arteries was 30–40% of cardiac and skeletal muscle (Park et al. 2014). Citrate synthase (CS) activity, an index of mitochondrial activity, in the human small arteries was 20–40% of human cardiac and skeletal muscle suggesting lower mitochondrial density. The authors concluded that the small artery smooth muscle had lower mitochondrial density with identical capacity for oxidative phosphorylation with other parameters suggestive of intrinsic functional differences between the different muscle groups. The RNASeq data here are consistent with this paradigm. CS mRNA was ∼2.5-fold higher in AO versus MA, very similar to the differences described above, while other mRNAs showed much larger differences in expression consistent with specific differences in metabolic pathways. While to the best of our knowledge energetic considerations in relation to contractile function of the small arteries has not received much investigation, a recent study has suggested that resistance as compared to conduit artery smooth muscle is more sensitive to by-products of oxidation such as reactive oxygen species due to lower expression of antioxidant enzymes (Burgoyne et al. 2012). The significance of the differing metabolic and mitochondrial expression profiles in MA versus AO with regard to disease processes, such as lipid dys-metabolism causing atherosclerosis in the large arteries, and ischemia causing contractile dysfunction in the small arteries, deserves further investigation.

### Signaling pathways

Arterial smooth muscle tone is highly controlled by a plethora of activating and inhibitory signaling pathways. The vasodilatory endothelial NOS was present at similar levels in MA and AO while Alox15 was almost 30-fold enriched in MA and in AO ∼30-fold less abundant than eNOS. Alox15 converts arachidonic acid to hydroperoxyeicosatetraenoic acid (HPETE), an endothelial-derived hyperpolarizing (relaxing) factor (Campbell and Gauthier 2013). This expression pattern is consistent with a greater functional role of EDHF versus NO (EDRF) in the control of tone in resistance versus conduit arteries, and may explain why prior studies have found it to be inducible in AO (Campbell and Gauthier 2013) but downregulated by endurance exercise in rat skeletal muscle feed arteries (Padilla et al. 2014). The vasoconstrictor *α*1a and *α*1d adrenergic receptor (Adra1a and 1d) mRNAs were nearly equally abundant and similar in MA and AO, consistent with their functional redundancy (Methven et al. 2009; Zacharia et al. 2013). The *α*2 receptors (Adra2a-c) were expressed at ∼10% of the level of *α*1s, consistent with their minor contribution to sympathetic vasoconstriction in most vascular beds (Zacharia et al. 2013) and their downregulation during postnatal maturation of rat mesenteric arterial smooth muscle (Reho et al. 2014a). The *β*-1 and *β*-2 adrenergic receptors were present in AO and MA at ∼1/5 the level of *α*-adrenergic receptors, consistent with their more minor and less certain role in sympathetically mediated vasorelaxation. *β*3 adrenergic receptor (*β*3AR) mRNA was highly abundant in AO and MA without differential expression. The role of *β*3AR in vascular relaxation is controversial; it is highly expressed in fat cells and also has been detected in endothelium and vascular adventitia (Flacco et al. 2013), and has been proposed to contribute to vasorelaxation through direct and/or paracrine mechanisms (Dessy and Balligand 2010; Balligand 2013). With regard to other sympathetically mediated signals, P2rx1 and P2rx5 were reciprocally expressed in MA and AO, respectively, consistent with prior studies, but the functional significance of their tissue-specific expression is unknown (reviewed in (Burnstock and Ralevic 2014)). In contrast in the third arm of sympathetic signaling, Neuropeptide Y (Zukowska 2005), Npy1R was the only member expressed in MA and AO and at moderately higher levels in the MA.

### Potentially novel signaling pathways

The unbiased sequencing of RNA pools identified a number of other receptors that are significantly or differentially expressed in MA versus AO and could be novel drug targets. Of particular interest was the moderate expression of several olfactory receptors. There is increasing appreciation of the expression of odorant and other chemosensors outside the nasal epithelium (Flegel et al. 2013) and their roles in the regulation of vascular (Pluznick et al. 2013) and airway smooth muscle (reviewed in (Pluznick 2013)). Rat Olr59 was highly restricted in its expression, being 10-fold more abundant in MA versus AO, and almost exclusively expressed in the mural compartment of the MA. This is consistent with a study of the pattern of expression of the mouse homolog Olfr78, shown to have as a ligand the small chain fatty acid propionate and to mediate mesenteric artery vasodilation (Pluznick et al. 2013). The vascular function of Olr63, the only other Olr significantly, but not differentially expressed, has not been examined.

### Genes not expressed or at very low levels

The sensitivity and specificity of deep sequencing of RNA samples and analysis of absolute rather than relative expression also provides valuable information by (1) excluding genes that are not significantly expressed, and (2) comparison of expression levels of gene products that are thought to regulate one another. With regard to transcriptional control, of the four skeletal myogenic bHLH differentiation factors (MyoD, myogenin, Myf5, Myf6), two were detected in only one of four samples (MA2) and at a very low level, likely reflecting low level skeletal muscle contamination. In contrast a prior RNASeq study reporting fold change suggested these mRNAs may be induced in skeletal muscle small arteries in a rat model of exercise and obesity (Jenkins et al. 2014; Padilla et al. 2014). With regard to contractile function, it has been suggested that IKK2 (IKBKB) may also function as a myosin light chain kinase (Ying et al. 2013). Its expression at levels ∼30-fold lower than MLCK and its substrate MLC20 would suggest that the stoichiometry is not appropriate for such function, at least in the MA. Similarly nonmuscle myosin has been suggested to contribute to force production in mouse aortic smooth muscle (Yuen et al. 2009), while its expression at ∼100-fold levels less than smMHC in MA makes it unlikely to contribute significantly to force production in this tissue. Lastly, single-nucleotide polymorphisms in Gnb3 have been associated with human heart failure and predict enhanced response to the vasodilator therapy with hydralazine and nitrates in the A-HeFT (African American Heart Failure Trial) (McNamara et al. 2014). RNASeq shows no significant expression of Gnb3 in rat MA or AO, while paralogs such as Gnb1, 2, 5 are highly expressed (data not shown). If also true in humans this would suggest either that the Gnb3 SNP is a marker without functional significance or alternatively, that its effect is mediated in a different tissue.

### Limitations and strengths of the current study

The use of whole tissue lysates as the source of RNA has advantages and disadvantages. As all of the cell types within the artery are represented, mRNAs expressed in each cell type may be identified. However, differential expression could reflect differences in cell populations between samples, and mRNAs from less abundant cell types such as endothelial cells are likely to be underrepresented within the samples. To address the first concern, we used a comparative approach amongst gene family members, genes with similar functions, and genes known to be specifically expressed in the different cell types within the vessel wall. We also validated a subset of mRNAs as enriched in purified endothelial versus mural cell populations in the MA. Ultimately validation of the cells of origin of the differentially expressed mRNAs will require a similar high throughput approach applied to highly enriched population of cells as could be done using mouse Cre-Lox technology to fluorescently label and purify cells. Purification of different cell populations from tissues can introduce its own bias including differential enrichment of cell sub-populations, continued contamination by other cell types, effect of the enrichment process on mRNAs and degradation of mRNAs during the enrichment process. Further the small size of the mesenteric arteries confounds many approaches used to study gene expression. As a distinct alternative approach, mRNA and protein expression may be determined in specific cell types by in situ histological assays though these are less quantitative.

While the RNASeq procedure is essentially unbiased, an element of bias is introduced by our recognition and selection of genes of interest. The pathway analyses provide some level of support for group differences between data sets but these recognition programs are still somewhat limited as well. By placing this data set in the public domain analyses by other investigators with their own biases may lead to alternative interpretations of the data. Having only a single replicate for each sample may have limited the ability to observe differences in the levels of some mRNAs between tissues, particularly those where the difference is closer to 2-fold (or less) but which could still be functionally important. Nonetheless with two samples in each group we identified through this screening process a number of mRNAs that are differentially expressed with high statistical probability. The high cost and time requirements for this analysis mandate thoughtful and parsimonious execution of these experiments. In the current study, mRNAs were not measured during arterial maturation, and it is possible that genes could be more highly expressed and play a greater role during developmental diversification of the vascular system.

A great deal of diversity is generated by differential exon usage including alternative transcriptional start sites within genes and alternative splicing of exons (Wang et al. 2008; Merkin et al. 2012). Differential exon usage is confounded by transcript abundance and requires more detailed analysis, a subject for future studies. We did find that Exon24 of PPP1r12a (Mypt1) was preferentially spliced in MA versus AO (data not shown), validating the RNASeq and our previous studies using other methods of measurement (Khatri et al. 2001; Payne et al. 2004, 2006; Zhang and Fisher 2007; Reho et al. 2014a; Zheng et al. 2015) (reviewed in (Dippold and Fisher 2014b; Fisher 2010)). The present study provides a description of the transcriptional gene program of the small resistance-type rat mesenteric arteries and contrasts it with that of a large conduit artery, the aorta. This provides a foundation for further study of the unique gene program of the small arteries, and the testing or discarding of hypotheses regarding the genetic and molecular basis of unique aspects of resistance artery function.

## Conflict of Interest

None declared.
